# Case of Dracuncules or Guinea-Worm

**Published:** 1845-08

**Authors:** 


*Case of Dracuncules or Guinea-Worm.*—Towards the close of the year 1843, a man, who had returned from Senegal, was admitted into the St. Antoine Hospital, at Paris, for a small furuncular swelling on the dorsum of the left foot. It had existed for about a month, and was accompanied with intolerable itching in the part. An incision was made upon it, and next day M. Maisonneuve observed that a white filiment projected from the wound: this was drawn out to the extent of about nine inches, and then it broke across. Another boil formed a little way below the head of the fibula; and as there was a tortuous, somewhat indurated, and painful subcutaneous line, extending from this point towards the calf of the leg, the skin was divided in two places, and an entire worm drawn out: it was of the thickness of a crow-quill, and upwards of two feet in length; it much resembled in appearance the *vas deferens.* The purulent matter from the two abscesses was carefully examined with the microscope; and, in that from the second one, there were observed myriads of minute animalcules, that moved about with great rapidity. On examining the worm itself, a milky fluid was perceived to be contained at one part of its tubular body.

In this fluid, too, numerous animalcules, similar to those which we have alluded to, were detected by the aid of the microscope. M.

Maisonneuve very reasonably concludes that these embryotic Dracunculi may readily insinuate themselves under the skin in persons, who are in the habit of walking about with naked feet in such countries as that of Senegal. There is therefore no occasion to have recourse to the very questionable doctrine of Spontaneous Generation to account for the development of these subcutaneous entozoa. It is probable that the worm, when developed under the integuments, remains quiet until the period for discharging its ova has arrived, and then it makes an effort to perforate the skin, and becomes liberated for that purpose.—*Ibid, from Arch. Gen. de Med.*

